# Can simple trachelectomy or conization show comparable survival rate compared with radical trachelectomy in IA1 cervical cancer patients with lymphovascular space invasion who wish to save fertility? A systematic review and guideline recommendation

**DOI:** 10.1371/journal.pone.0189847

**Published:** 2018-01-31

**Authors:** Seung-Hyuk Shim, Myong Cheol Lim, Hyun Jung Kim, Maria Lee, Eun Ji Nam, Jung Yun Lee, Yoo-Young Lee, Kwang Beom Lee, Jeong Yeol Park, Yun Hwan Kim, Kyung Do Ki, Yong Jung Song, Hyun Hoon Chung, Sunghoon Kim, Jeong-won Lee, Jae-Weon Kim, Duk-Soo Bae, Jong-Min Lee

**Affiliations:** 1 Department of Obstetrics and Gynecology, Konkuk University School of Medicine, Seoul, Korea; 2 Cancer Healthcare Research Branch, Center for Uterine Cancer, and Center for Clinical Trials, Research Institute and Hospital, Department of Cancer Control and Population Health, Graduate School of Cancer Science and Policy, National Cancer Center, Ilsan-ro, Ilsandong-gu, Goyang-si Gyeonggi-do, Republic of Korea; 3 Department of Preventive Medicine, College of Medicine, Korea University, Seoul, Korea; 4 Department of Obstetrics and Gynecology, Seoul National University College of Medicine, Seoul, Korea; 5 Department of Obstetrics and Gynecology, Yonsei University College of Medicine, Seoul, Korea; 6 Division of Gynecologic Oncology, Princess Margaret Cancer Centre, University Health Network, University of Toronto, Toronto, Canada; 7 Department of Obstetrics and Gynecology, Gachon University Gil Medical Center, Gachon University College of Medicine, Incheon, Korea; 8 Department of Obstetrics and Gynecology, University of Ulsan College of Medicine, Asan Medical Center, Seoul, Korea; 9 Department of Obstetrics and Gynecology, Ewha Womans University Mokdong Hospital, Ewha Womans University School of Medicine, Seoul, Korea; 10 Department of Obstetrics and Gynecology, Kyung Hee University Hospital at Gangdong, Kyung Hee University College of Medicine, Seoul, Korea; 11 Department of Obstetrics and Gynecology, Pusan National University School of Medicine, Pusan, Korea; 12 Department of Obstetrics and Gynecology, Samsung Medical Center, Sungkyunkwan University School of Medicine, Seoul, Korea; National Academy of Medical Sciences, NEPAL

## Abstract

**Objective:**

This study aims to analyze the published literatures on the effect of less radical fertility-preserving procedures, such as conization or simple trachelectomy, on oncological outcomes in IA1 cervical cancer patients with lymphovascular space invasion (LVSI) through a systematic-review.

**Methods:**

The EMBASE and MEDLINE databases and Cochrane Library were searched for published studies reporting the oncological outcomes of conization/simple trachelectomy in these patients, through April 2017. The endpoints were recurrence and mortality rates. Data were presented as per the Meta-analysis Of Observational Studies in Epidemiology checklist. Practice guidelines were generated via the Grading of Recommendation, Assessment, Development and Evaluation system.

**Results:**

From 6,755 records, 94 full-texts articles were reviewed for eligibility, and five studies were included in this systematic review. All included studies were nonrandomized studies: two case-control studies comparing conization (n = 14) with hysterectomy (n = 24), and the other three were interrupted time series including conization (n = 20) and simple vaginal trachelectomy (n = 59). During the median follow-up duration of 43 months, no recurrence was reported in both conization and simple trachelectomy groups in IA1 patients with LVSI. From three studies reporting the fertility outcomes, the rates of pregnancy, live birth, preterm delivery, and second-trimester miscarriage were 73% (35/48), 64% (32/50), 10% (5/48), and 6% (3/48), respectively.

**Conclusion:**

Results suggest that simple trachelectomy or conization could be performed for IA1 cervical cancer patients with LVSI who want to preserve fertility, although these results are only based on a small number of nonrandomized studies (recommendation grade 2 = weak; evidence level D = very low). Further randomized trials with long-term study period are needed to address this issue.

## Introduction

Cervical cancer is the fourth most common cancer in women; 528,000 new cases were diagnosed in 2012 [1]. In Korea, cervical cancer incidence has been decreasing due to effective screening, with annual percent change of −4.3% recently [2]. The age-standardized incidence rate is 9.5 per 100,000 persons in 2013 [3]. However, cervical cancer remains to be the most common gynecologic cancer, and its incidence is still increasing in young women aged <30 years, with annual percent change of +4.8% [4]. Traditionally, a widespread concept that cervical cancer is not a disease of young women exists. However, according to the 2009–2013 Surveillance, Epidemiology, and End Results data, 38% of cervical cancer cases are diagnosed in women <45 years old [1]. Similarly, cervical cancer incidence in Korea has increased in women <35 years old from 2001 to 2011 [5]. With the highlight on the quality of life, preserving fertility is a crucial issue for the treatment of these reproductive-age patients.

The International Federation of Gynecology and Obstetrics (FIGO) stage IA accounts for 25% of cervical cancers, and 85% of stage IA diseases are stage IA1 [2]. Of note, 50% of patients with IA cervical cancer are under 40 years old [6]. The diagnosis of stage IA1 can only be established via microscopic evaluation of lesion, typically with conization. The depth of stroma invasion should be <3 mm and the horizontal spread <7 mm. Lymphovascular space invasion (LVSI) does not change the FIGO staging but should be separately reported because it may affect treatment strategies. The treatment options for stage IA1 disease are determined based on the desire of fertility preservation and the LVSI status. The current practice guidelines uniformly state therapeutic strategies for IA1 patients who do not desire to preserve fertility: simple/extrafascial hysterectomy (Type A) is recommended for stage IA1 patients without LVSI, and modified radical hysterectomy (Type B) and pelvic lymphadenectomy are preferred for IA1 patients with LVSI [6–8]. In case of fertility preservation, conization (with negative margins) is an option in stage IA1 patients without LVSI. Meanwhile, the therapeutic options for fertility preservation vary according to the guidelines in stage IA1 patients with LVSI; the NCCN guidelines recommend radical trachelectomy with pelvic lymphadenectomy, and conization with pelvic lymphadenectomy as the alternative [8]. The ESMO guidelines recommend conization with pelvic lymphadenectomy [7]. The Japanese Society of Gynecologic Oncology (JSGO) guidelines do not comment on this issue [6].

All these options have not been compared in randomized clinical trials. In the absence of level I evidence, the physicians’ personal interpretation of published results might strongly influence the choice of fertility-preserving procedures. In this study, we review the oncological and fertility outcomes of less radical fertility-preserving procedures, such as conization or simple trachelectomy, for the treatment of stage IA1 cervical cancer with LVSI and assess whether these options show comparable survival outcome compared with radical trachelectomy in those patients. The Korean Society of Gynecologic Oncology (KSGO) recently revised the practice guidelines for cervical cancer and selected nine key questions arising in clinical situations; these questions were derived from thorough discussions with diverse experts in radiology, pathology, medical oncology, radiation oncology, and nuclear medicine. This topic is one of the nine selected key questions.

## Materials and methods

### Literature search

A systematic review was performed using the designed reporting guidelines (S1 Appendix) [9, 10]. The EMBASE and MEDLINE databases and Cochrane Central Register for Controlled Trials were searched up to April 2017, irrespective of language. Prepublication papers were also reviewed. The search strategy is described in the S2 Appendix. Titles and abstracts were screened to identify relevant articles, and full texts were retrieved for detailed reviews. References in retrieved papers and review articles were manually checked to find additional relevant studies. Three authors (SS, ML, and HK) independently performed all search.

### Eligibility criteria

Inclusion criteria for this systematic review were as follows: (1) randomized controlled trial (RCT) or prospective or retrospective cohort, nested case control, or population-based case control study or interrupted time series (ITS) that fulfilled the minimum criteria given by the Cochrane Effective Practice and Organisation of Care Group (EPOC) [11]; (2) participants with stage IA1 with LVSI receiving fertility-preserving surgery; (3) conization or simple trachelectomy as the intervention of interest; and (4) outcome measure of recurrence or mortality rate measured via relative risks, odds ratios, or hazard ratios with 95% confidence intervals (or sufficient data for calculation). For studies with shared or duplicated data, the most recent or informative study was selected.

### Data extraction

The following data were obtained from eligible studies: name of the authors; publication year; study design, location, and period; age; sample size, details of fertility-preserving surgery; tumor characteristics (FIGO stage; tumor size, histology, and LVSI status); follow-up duration; morbidity related to treatment; oncologic outcomes (recurrence and death from disease); fertility outcomes; and variables controlled for the analysis. Each study was systematically reviewed for features that might introduce bias, similarity of risk factors for prognosis, and follow-up in conization/simple trachelectomy groups. Three authors (SS, ML, and HK) independently extracted data with the use of a standard extraction sheet; discrepancies were jointly reviewed until consensus was reached.

### Quality assessment

For nonrandomized studies (NRSs), the quality of each study was evaluated using the nine-star Newcastle–Ottawa Scale (NOS) in three categories: selection, comparability, and exposure (case-control studies) or outcomes (cohort studies) [12]. A study receiving five or more stars was defined as high quality [13]. To evaluate the risk of bias for ITS studies, the seven standard criteria suggested by EPOC are used [14]. Two authors (SS and HK) independently evaluated the study quality and resolved any disagreement after discussion with all the other authors.

### Developing practice guidelines

We made an “evidence profile” for the quality of evidence based on the Grading of Recommendation, Assessment, Development and Evaluation (GRADE) system [15]. The system considered study qualities, such as consistency, directness of evidence, and the methodologic quality, to rate an overall quality of the supporting evidence into four categories (A, high; B, moderate; C, low; D, very low). Finally, we developed the practice guidelines incorporating the risks and benefits of the compared interventions, provided with the strength of the recommendation (1, strong; 2, weak) and the quality of the supporting evidence (A, high; B, moderate; C, low; D, very low).

A consensus-building meeting was conducted for the revised guidelines and key questions by the KSGO Executive Committee on October 2015. The levels of recommendations were determined by voting by the KSGO members and were passed if 50% or more of the total votes were in favor. Subsequently, the review and guidelines were presented at the 21^st^ Annual Symposium of KSGO on November 2015.

## Results

### Literature search

**Fig 1:**
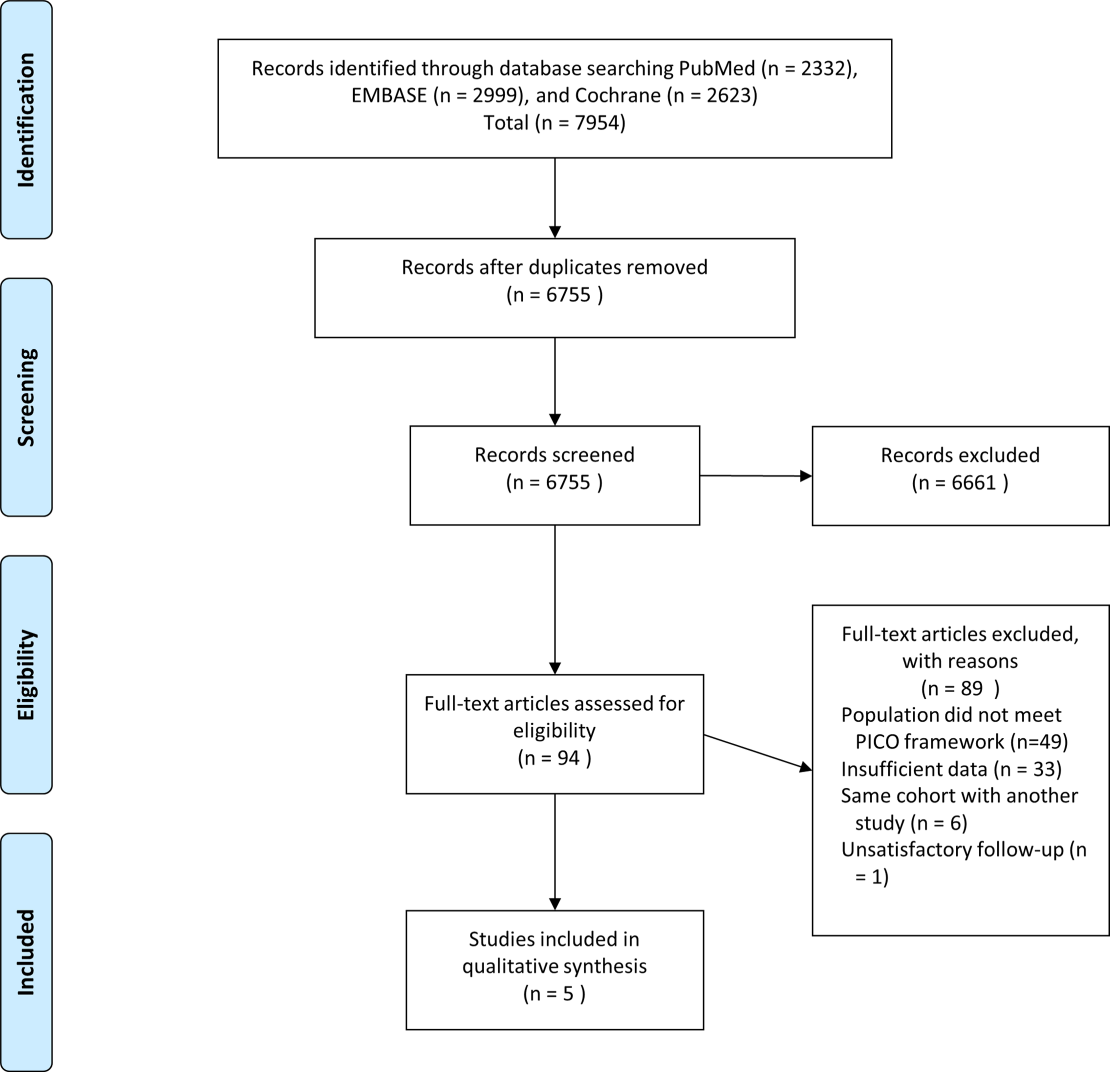
Flow diagram of the literature search procedure.

Fig 1 presents a flow diagram of relevant study identification. From 6,755 records, 94 potentially relevant papers were identified, and full texts were reviewed for eligibility. Forty-nine did not meet the PICO framework. Thirty-three were incomplete studies that provided recurrence/mortality data, one showed unsatisfactory follow-up, and six were studies involving duplicated data used for other studies. These were excluded from further analysis. Five studies were therefore included in this systematic review [16–20]. S1 Table shows the excluded studies with reasons for exclusion.

### Study characteristics and details

Table 1 lists the detailed study characteristics. Five papers involving 59 IA1 patients with LVSI undergoing conization or simple trachelectomy and 24 IA1 patients with LVSI undergoing hysterectomy were published between 2002 and 2014. Studies were conducted in the Netherlands [17], Korea [18], US [16], Czech Republic [19], and Canada [20]. All included studies were nonrandomized observational studies: two were retrospective case-control studies [17, 18], and three were ITS studies [16, 19, 20]. The quality scores for the two case-control studies were seven on the nine-star NOS (S2 Table). The risk of bias for the three ITS studies are shown according to the seven standard criteria by EPOC in S3 Table.

**Table 1:** Characteristics of studies included in the systematic review.

Study/design	Location/study period	Treatment		Recurrence		Death		Mean age (yr)	Stage	LVSI	Histology (n)	PLND	RM after conization	Mean follow-up period (Mo)
		Intervention (n)	Control (n)	Intervention (n)	Control (n)	Intervention (n)	Control (n)							
Bekkers et al. ^(17)^/ Retrospective case-control study	Netherlands/1981–1999	Conization (5)	Hysterectomy (4)	0	0	0	0	34	IA1 (9)	Presence	Squamous (9)	No	Negative	72*
Lee et al. ^(18)^/ Retrospective case-control study	Korea/1997–2006	Conization (9)	Hysterectomy (20)	0	0	0	0	35	IA1 (29)	Presence	Squamous (29)	No	Negative	34
Plante et al. ^(20)^/ Prospective single arm cohort study	Canada/2007–2016	Simple vaginal trachelectomy (35)	NA	1^†^	NA	0	NA	29*	IA1 (8), IA2 (9), IB1 (18)	Presence^‡^	Squamous (19) Adenocarcinoma (13) Adenosquamous (1) Clear cell (1) Undifferentiated (1)	Yes (SLN mapping + PLND 28, SLN mapping alone 7)	Negative	48
Andikyan et al. ^(16)^/ Retrospective single arm cohort study	US/2005–2012	Conization (10)	NA	0	NA	0	NA	28*	IA1 (7), IB1 (3)	Presence	Squamous (8) Adenocarcinoma (1) Clear cell (1)	Yes (SLN mapping 10)	Negative	17*
Rob et al. ^(19)^/ Retrospective single arm cohort study	Czech Republic/1999–2006	Conization (10) Simple vaginal trachelectomy (24)*	NA	1^§^	NA	0	NA	28	IA1 (3) IA2 (10) IB1 (27)	Presence^∥^	Squamous (32) Adenocarcinoma (7) Adenosquamous (1)	Yes (SLN mapping 40)	Negative	47*
All combined		Conization (34) Simple vaginal trachelectomy (59)*	Hysterectomy (24)	2^¶^	0	0	0	30**						43**

LVSI, lymphovascular space invasion; PLND, pelvic lymph node dissection; RM, resection margin; NA, not applicable; SLN, sentinel lymph node

*Median

^†^Patient details: stage IB1, invasion 6 mm, extension measuring 16 mm with LVSI, 2 SLNs with isolated tumor cells.

^‡^There were 8 stage IA1 patients (100% with LVSI).

^§^Patient details: stage IB1, conization invasion 8 mm, extension measuring 7 mm with LVSI, 27 negative nodes, subsequent trachelectomy.

^∥^There were 3 stage IA1 patients (100% with LVSI), 10 IA2 patients (40% with LVSI), and 27 IB1 patients (38.5% with LVSI).

^¶^Overall, no recurrence occurred in patients with IA1 disease.

**Weighted average

The two case-control studies compared conization (n = 14) with hysterectomy (n = 24). All included patients had FIGO stage IA1 with LVSI. All the patients with involved resection margin after the first conization received repeat conization to ensure negative resection margin. The histologic type was squamous cell only, and lymphadenectomy was not performed in these studies. During the follow-up period, which was longer than 30 months in these studies, no recurrence was reported in both conization and hysterectomy groups.

The three ITS studies included conization (n = 20) and simple vaginal trachelectomy (n = 59). Of the included patients, 18 had FIGO stage IA1 with LVSI, and 51 had IA2 or IB1. The histologic types were squamous cell (n = 59) or adenocarcinoma (n = 20). All the patients with involved resection margin after first conization received repeat conization to ensure negative resection margin. Laparoscopic lymphadenectomy including sentinel lymph node (SLN) mapping was performed in all ITS studies, and two patients were found to have lymph node involvement. During the follow-up period, which was longer than 17 months in these studies, two recurrences were reported. One patient initially received simple trachelectomy for stage IB1 cervical cancer with LVSI (invasion, 6 mm; diameter, 16 mm; two SLNs with isolated tumor cells)[20]. This patient was treated with chemoradiation for central recurrence, and no evidence of disease was noted at the time of publication. The other patient initially received simple trachelectomy for stage IB1 cervical cancer with LVSI (invasion, 8 mm; diameter, 7 mm; 27 negative nodes)[19]. This patient was treated with chemoradiation therapy for central recurrence, and no evidence of disease was noted 60 months later. Overall, no recurrence occurred in patients with IA1 disease.

Three studies reported the fertility outcomes (Table 2)[18–20]. Of those, one reported only live birth rate of 100% (2 out of 2)[18], and two reported both pregnancy rates and live birth rates [19, 20]. Rob et al. reported a pregnancy rate of 71% (17 out of 24) and live birth rate of 52% (12 out of 23, 3 pregnancies ongoing at the time of publication)[19]. Meanwhile, Plante et al. reported a pregnancy rate of 75% (18 out of 24) and live birth rate of 72% (18 out of 25)[20]. Overall, from three studies reporting the fertility outcomes, the rates of pregnancy, live birth, preterm delivery, and second-trimester miscarriage were 73% (35/48), 64% (32/50), 10% (5/48), and 6% (3/48), respectively.

**Table 2:** Fertility outcomes of studies included in the systematic review.

Study/design	Fertility outcomes					
	Attempting to Conceive, n (%)	Pregnancy rate, n (%)	Live birth rate, n (%)	Preterm delivery, n (%)	First trimester fetal loss, n (%)	Second trimester fetal loss, n (%)
Bekkers et al. ^(17)^/ Retrospective case-control study	NR	NR	NR	NR	NR	NR
Lee et al. ^(18)^/ Retrospective case-control study	NR	NR	2/2 (100)	NR	NR	NR
Plante et al. ^(20)^/ Prospective single arm cohort study	24/35 (69)	18/24 (75)	18/25 (72)	2/25 (8)	5/25 (20)	0/25 (0)
Andikyan et al. ^(16)^/ Retrospective single arm cohort study	NR	NR	NR	NR	NR	NR
Rob et al. ^(19)^/ Retrospective single arm cohort study	24/32 (75)	17/24 (71)	12*/23 (52)	3^†^/23 (13)	5^‡^/23 (22)	3/23 (13)
All combined	48/67 (72)	35/48 (73)	32/50 (64)	5/48 (10)	10/48 (21)	3/48 (6)

LVSI, lymphovascular space invasion; NR, not reported

*Three pregnancies ongoing at the time of publication.

^†^In three of these cases, premature delivery occurred (24th, 34th, and 36th weeks).

^‡^Two women decided on elective abortion for personal reason; one woman was diagnosed with extrauterine pregnancy; one woman miscarried twice in the first trimester.

### Developing practice guidelines

In the practice guidelines for cervical cancer recently revised by KSGO [21], the following question was selected as nine key questions arising in clinical situations: “Does simple trachelectomy or conization have similar survival outcome with radical trachelectomy in cervical cancer stage IA1 patients with LVSI who want to preserve fertility?” The following recommendation was suggested by the KSGO Executive Committee: “Simple trachelectomy or conization could be performed for cervical cancer IA1 patients with LVSI who want to preserve fertility, based on the similar survival outcomes from radical trachelectomy (Recommendation grade 2 = weak; Evidence level D = very low).” The “evidence profile” according to the GRADE system is shown in S4 Table.

## Discussion

In this study, we systematically reviewed oncological outcomes of conization or simple trachelectomy in stage IA1 cervical cancer patients with LVSI. Although no study is available to compare conization or simple trachelectomy with radical trachelectomy, the published data so far reported that no recurrence was observed in those patients after conization or simple trachelectomy. Thus, these fertility-preserving procedures could be performed for cervical cancer IA1 patients with LVSI who want to preserve fertility based on the current systematic review, albeit the results are based on small number of nonrandomized studies (Recommendation grade 2 = weak; Evidence level D = very low). Before deciding on fertility preservation, a thorough examination by specialized gynecologic pathologists is essential to accurately evaluate the histologic type, depth of stromal invasion, and the status of resection margin and LVSI.

Since Daniel Dargent first introduced the vaginal radical trachelectomy and laparoscopic lymphadenectomy to preserve fertility in patients with cervical cancer in 1986 [22], it has been the standard fertility-preserving treatment in cases of FIGO stage IA1 with LVSI, IA2, and IB1 with tumor <2 cm [6–8, 23–25]. However, resection of the parametrium and removal of normal cervical stroma beyond the tumor during radical trachelectomy could compromise fertility [26]. Indeed, approximately 30% of the pregnancies in women undergoing laparoscopic radical trachelectomy ended in miscarriage or preterm birth [26, 27]. Moreover, radical trachelectomy may cause adverse surgical outcomes in terms of urologic and neurologic morbidities [28]. To overcome these caveats of radical trachelectomy, less radical fertility-sparing approaches without parametrial resection, such as conization or simple trachelectomy, have been advocated. In 2007, Rob et al. first reported these approaches incorporating two steps without compromising the oncologic outcome: first, laparoscopic SLN dissection and second, conization for IA1 with LVSI and IA2 or simple trachelectomy for IB1 with the tumor less than 2 cm if the SLNs are negative [29]. In their updated published data of 40 enrolled patients, 34 received conization or simple trachelectomy (six with positive SLNs received radical hysterectomy) [19]. During the median follow-up duration of 47 months, only one patient who had IB1 with LVSI had recurrence, and none of the 13 patients with IA1 with LVSI or IA2 had recurrence.

Several studies have reported a low risk of parametrial involvement in low-risk early cervical cancer with favorable clinicopathological variables (i.e., tumor size ≤2 cm, <50% stromal invasion on MRI, negative LVSI, negative LNM, and depth of stromal invasion <10 mm on conization) [30–34]. In the review by Ramirez et al., two out of the 247 patients who received the less radical surgery without parametrial resection for low-risk early cervical cancer had recurrence [35]. These findings support that less radical surgeries without parametrial resection are applicable for the selected patients. In the same context, less radical fertility-sparing surgery, such as conization or simple trachelectomy, can be provided to those patients. It has been reported that 26% of young patients with early-stage cervical cancer treated with radical hysterectomy may have been eligible for fertility-sparing surgery, such as conization [36]. When considering less radical fertility-sparing surgery for IA1 with LVSI, the crucial question is whether isolated LVSI can be an independent predictor for parametrial involvement in the absence of other risk factors (i.e., tumor size ≤2 cm, <50% stromal invasion on MRI, negative lymph node metastasis, and depth of stromal invasion <10 mm on conization). Interestingly, the published studies so far uniformly reported no parametrial involvement in stage IA1 patients who have LVSI but negative lymph node metastasis [33, 34, 37–39]. Therefore, conization or simple trachelectomy can be a reasonable fertility-sparing procedure for IA1 with LVSI if negative lymph node metastasis is guaranteed.

Of the five studies included in the present systematic review, pelvic lymphadenectomy was not performed for IA1 with LVSI in two studies [17, 18]. Although no lymph node recurrence was found during the study period in both the two studies, the number of patients is too small to support omitting lymphadenectomy in this group. In the other three studies, pelvic lymphadenectomy including SLN mapping was routinely performed. In the study by Rob et al., six IB1 patients were excluded for fertility-sparing surgery due to positive lymph node metastasis after SLN mapping [19]. Current guidelines uniformly recommend performing pelvic lymphadenectomy in IA1 with LVSI patients [7, 8] because LVSI status has been considered as a surrogate for lymph node metastasis [35]. Ensuring the negative parametrial involvement (as per the aforementioned rationale) as well as being consistent with those guidelines, we recommend performing lymphadenectomy for IA1 with LVSI. Given the low rate of lymph node metastasis in this group, SLN mapping can be an option to avoid complete lymphadenectomy and the related adverse events.

Regarding the fertility outcomes, a pregnancy rate ranged from 71% to 75%, although second-trimester miscarriage rate increased compared with the general population (6% vs 1.6%) [40, 41]. However, those results are still superior to what have been reported after radical trachelectomy [26]. A recent review on various fertility-sparing procedures for cervical cancer confirmed a higher rate of miscarriage (21% vs 14%) and of preterm delivery (21% vs 12%) for vaginal radical trachelectomy compared with simple trachelectomy or conization [27]. The important factor in second-trimester miscarriage or preterm delivery is the amount of residual cervical stromal tissue after fertility-sparing procedures [42]. In this regard, the fertility outcomes after conization or simple trachelectomy must be better because these procedures offer a larger amount of cervical stroma compared with radical trachelectomy. In most series, a cerclage was not routinely performed; however, 24 out of the 35 simple trachelectomy cases in the study by Plante et al. received the cerclage [20]. Although the rates of second-trimester miscarriage and preterm delivery in the study by Plante et al. were lower than those in the simple trachelectomy series by Rob et al., whether routine cerclage would improve the fertility outcomes remains unclear. Some investigators were concerned that cerclage may negatively affect fertility due to cervical erosion, stenosis, and chronic vaginal discharges [43]. Thus, consensus for routine cerclage has not been reached at this point, and we recommend individualized application considering the residual cervical length.

The main strength of the present study is its team-based approach to a comprehensive literature review and a standardized process for reviewing the evidence and generating the guidelines. These processes allowed us to summarize all relevant studies and generate helpful information for both clinicians and patients in decision making for less radical fertility-preserving procedures for IA1 cervical cancer with LVSI.

Our study had also several limitations, and thus the results should be interpreted with caution. First, all the included studies were NRS. This feature may impede the comprehensive reporting of any confounding factors. Second, the total number of enrolled cases is small. Therefore, the data were insufficient to execute meta-analysis or draw definite conclusions. Third, the follow-up duration was not long enough to ensure the long-term oncological outcomes with the weighted mean periods of included studies of 43 months. For these reasons, the strength of recommendation was determined as weak and the level of evidence as very low in the practice guidelines for cervical cancer by KSGO [21].

## Conclusions

In conclusion, the results suggest that simple trachelectomy or conization could be performed for cervical cancer IA1 patients with LVSI who want to preserve fertility, although the results are based on a small number of NRSs subject to a variety of biases. RCTs with a long-term study period are ideal to address this issue. However, it is likely not feasible to conduct such an RCT because of the relative rarity and very low rate of recurrence in these patients. Two prospective observational trials are currently underway to assess the effect of conization and pelvic lymphadenectomy in low-risk early cervical cancer, including IA1 with LVSI [43, 44], and these trials will provide more solid evidence on the role of this strategy in these patients.

## Supporting information

S1 Appendix PRISMA checklist. (DOC)

S2 Appendix Supplemental methods. (DOC)

S1 Table Excluded studies with reasons. (DOCX)

S2 Table Newcastle–Ottawa Scale for the risk of bias and the quality assessment of included studies. (DOC)

S3 Table Seven standard criteria suggested by the Cochrane Effective Practice and Organisation of Care (EPOC) to evaluate the study quality for single-arm cohort studies. (DOC)

S4 Table An “evidence profile” by grading the quality of evidence according to the Grading of Recommendation, Assessment, Development and Evaluation (GRADE) system. (DOC)

S1 File All relevant data anonym. (XLSX)
