# Supplementary material for: Can simple trachelectomy or conization show comparable survival rate compared with radical trachelectomy in IA1 cervical cancer patients with lymphovascular space invasion who wish to save fertility? A systematic review and guideline recommendation
Source: PLoS One. 2018 Jan 31;13(1):e0189847. doi: 10.1371/journal.pone.0189847 (PMC5791938; doi:10.1371/journal.pone.0189847)
Supplement: S2 Appendix — (DOC) [file pone.0189847.s002.doc]

**DATA SUPPLEMENTS**

**Supplemental Methods**

MEDLINE

1. "Uterine Cervical Neoplasms"[Mesh] 66554

2. "Adenocarcinoma"[Mesh:NoExp] OR "Adenocarcinoma, Clear Cell"[Mesh] OR "Adenocarcinoma, Mucinous"[Mesh] OR "Adenocarcinoma, Papillary"[Mesh] OR "Carcinoma, Endometrioid"[Mesh] OR "Carcinoma, Signet Ring Cell"[Mesh] OR "Carcinoma, Neuroendocrine"[Mesh:NoExp] OR "Carcinoma, Adenosquamous"[Mesh] OR "Carcinoma, Large Cell"[Mesh] OR "Carcinoma, Small Cell"[Mesh] OR "Carcinoma, Squamous Cell"[Mesh:NoExp] 266885

3. cervix[tiab] OR Cervical[tiab] OR "Signet Ring Cell"[tiab] OR Neuroendocrine[tiab] OR "Cervix Uteri"[Mesh] 266426

4. cancer[tiab] OR malignant[tiab] OR carcinoma[tiab] OR neoplasm[tiab] OR cancers[tiab] OR malignancy[tiab] OR carcinomas[tiab] OR neoplasms[tiab] 2104711

5. Adenocarcinoma[tiab] OR Adenocarcinomas[tiab] Adenosquamous[tiab] OR "Squamous Carcinoma"[tiab] OR "Squamous Carcinomas"[tiab] OR "Epidermoid Carcinoma"[tiab] OR "Epidermoid Carcinomas"[tiab] 11970

6. 2 OR 4 OR 5 2154726

7. 6 AND 3 103452

8. 7 OR 1 120810

9. "Conization"[Mesh] OR conization[tiab] OR trachelectomy[tiab] OR Conizations[tiab] OR Conisation[tiab] OR Conisations[tiab] OR trachelectomies[tiab] 2858

10. 8 AND 9 2332

EMBASE

1. 'uterine cervix tumor'/exp 101902

2. 'adenocarcinoma'/de OR 'adenoid cystic carcinoma'/exp OR 'adenosquamous carcinoma'/exp OR 'clear cell carcinoma'/exp OR 'large cell carcinoma'/exp OR 'small cell carcinoma'/exp OR 'undifferentiated carcinoma'/exp OR 'endometrioid carcinoma'/exp OR 'signet ring carcinoma'/exp OR 'large cell neuroendocrine carcinoma'/exp 143583

3. cervix:ab,ti OR Cervical:ab,ti 270909

4. "Signet Ring Cell":ab,ti OR Neuroendocrine:ab,ti OR cancer:ab,ti OR malignant:ab,ti OR carcinoma:ab,ti OR neoplasm:ab,ti OR cancers:ab,ti OR malignancy:ab,ti OR carcinomas:ab,ti OR neoplasms:ab,ti OR Adenocarcinoma:ab,ti OR Adenocarcinomas:ab,ti OR Adenosquamous:ab,ti OR "Squamous Carcinoma":ab,ti OR "Squamous Carcinomas":ab,ti OR "Epidermoid Carcinoma":ab,ti OR " Epidermoid Carcinomas":ab,ti 2783693

5. 2 OR 4 2799343

6. 3 AND 5 109585

7. 6 OR 1 141451

8. conization:ab,ti OR trachelectomy:ab,ti OR Conizations:ab,ti OR Conisation:ab,ti OR Conisations:ab,ti OR trachelectomies:ab,ti 3799

9. 'uterine cervix conization'/exp 2404

10. 8 OR 9 4446

11. 7 AND 10 3549

12. NOT ('editorial'/it OR 'letter'/it OR 'note'/it OR 'short survey'/it) 3214

13. NOT 'nonhuman'/de 2999

Cochrane Central Register for Controlled Trials

1. MeSH descriptor: [Uterine Cervical Neoplasms] explode all trees 1771

2. MeSH descriptor: [Adenocarcinoma] this term only 2444

3. MeSH descriptor: [Adenocarcinoma, Clear Cell] explode all trees 31

4. MeSH descriptor: [Adenocarcinoma, Mucinous] explode all trees 69

5. MeSH descriptor: [Adenocarcinoma, Papillary] explode all trees 23

6. MeSH descriptor: [Carcinoma, Endometrioid] explode all trees 32

7. MeSH descriptor: [Carcinoma, Signet Ring Cell] explode all trees 6

8. MeSH descriptor: [Carcinoma, Adenosquamous] explode all trees 38

9. MeSH descriptor: [Carcinoma, Large Cell] explode all trees 73

10. MeSH descriptor: [Carcinoma, Small Cell] explode all trees 747

11. MeSH descriptor: [Carcinoma, Neuroendocrine] this term only 3

12. MeSH descriptor: [Carcinoma, Squamous Cell] this term only 2136

13. 2-12/or 4835

14. cancer or malignant or carcinoma or neoplasm or cancers or malignancy or carcinomas or neoplasms or Adenocarcinoma or Adenocarcinomas Adenosquamous or "Squamous Carcinoma" or "Squamous Carcinomas" or "Epidermoid Carcinoma" or "Epidermoid Carcinomas":ti,ab,kw 89828

15. 13 or 14 89828

16. MeSH descriptor: [Cervix Uteri] explode all trees 968

17. cervix or Cervical or "Signet Ring Cell":ti,ab,kw 10584

18. 16 or 17 10584

19. 18 and 15 3276

20. 19 or 1 3276

21. 20/trials 2623
