# Supplementary material for: Can simple trachelectomy or conization show comparable survival rate compared with radical trachelectomy in IA1 cervical cancer patients with lymphovascular space invasion who wish to save fertility? A systematic review and guideline recommendation
Source: PLoS One. 2018 Jan 31;13(1):e0189847. doi: 10.1371/journal.pone.0189847 (PMC5791938; doi:10.1371/journal.pone.0189847)
Supplement: S1 Table — (DOCX) [file pone.0189847.s003.docx]

| **Article** | **Reason for Exclusion** |
| --- | --- |
| ([1-53](#_ENREF_1)) | Did not meet the PICO framework |
| ([54-86](#_ENREF_54)) | Did not provide exact recurrence/mortality data |
| ([87](#_ENREF_87)) | Unsatisfactory follow-up |
| ([88-93](#_ENREF_88)) | Duplicated data |
| ([94-96](#_ENREF_94)) | Review articles |

**S1 Table. Excluded studies and reasons for exclusion**

**References**

1. Abu-Rustum NR, Sonoda Y. Fertility-sparing surgery in early-stage cervical cancer: Indications and applications. JNCCN Journal of the National Comprehensive Cancer Network. 2010;8(12):1435-8.

2. Andersen ES. Laser conization in the management of cervical intraepithelial neoplasia and microinvasive carcinoma of the uterine cervix. Journal of Gynecologic Surgery. 2000;16(1):1-23.

3. Bisseling KCHM, Bekkers RLM, Rome RM, Quinn MA. Treatment of microinvasive adenocarcinoma of the uterine cervix: A retrospective study and review of the literature. Gynecologic Oncology. 2007;107(3):424-30.

4. Costa S, Marra E, Martinelli GN, Santini D, Casadio P, Formelli G, et al. Outcome of conservatively treated microinvasive squamous cell carcinoma of the uterine cervix during a 10-year follow-up. International Journal of Gynecological Cancer. 2009;19(1):33-8.

5. Dargent D, Beau GE, Frobert JL. Micro-invasive cancer of the uterine cervix: Results after conization. Gynecologie. 1982;33(1-2):71-6.

6. Ditto A, Martinelli F, Bogani G, Fischetti M, Di Donato V, Lorusso D, et al. Fertility-sparing surgery in early-stage cervical cancer patients: Oncologic and reproductive outcomes. International Journal of Gynecological Cancer. 2015;25(3):493-7.

7. Fagotti A, Gagliardi ML, Moruzzi C, Carone V, Scambia G, Fanfani F. Excisional cone as fertility-sparing treatment in early-stage cervical cancer. Fertility and Sterility. 2011;95(3):1109-12.

8. Gien LT, Covens A. Fertility-sparing options for early stage cervical cancer. Gynecologic Oncology. 2010;117(2):350-7.

9. Gleeson N, Gough F, Walsh CB, Turner M, Clinch J, Prendiville W. Large loop excision of the transformation zone (LLETZ) and LLETZ conisation in the management of early squamous carcinoma of the cervix. Journal of Obstetrics and Gynaecology. 1996;16(6):525-8.

10. He Y, Wu YM, Zhao Q, Wang T, Wang Y, Kong WM, et al. Clinical value of cold knife conization as conservative management in patients with microinvasive cervical squamous cell cancer (stage IA1). International Journal of Gynecological Cancer. 2014;24(7):1306-11.

11. Hefler LA, Polterauer S, Schneitter A, Concin N, Hofstetter G, Bentz E, et al. Repeat surgery in patients with cervical cancer stage FIGO IA1: a series of 156 cases and a review of the literature. Anticancer Res. 2010;30(2):565-8.

12. Itsukaichi M, Kurata H, Matsushita M, Watanabe M, Sekine M, Aoki Y, et al. Stage Ia1 cervical squamous cell carcinoma: Conservative management after laser conization with positive margins. Gynecologic Oncology. 2003;90(2):387-9.

13. Kabayama N, Uchida N, Togami S, Yokomine D, Kamio M, Yoshinaga M, et al. Clinical outcome of stage IA1 squamous cell carcinoma of the uterine cervix and pathological findings of initial conization. International Journal of Gynecological Cancer. 2012;22:E781.

14. Karimi-Zarchi M, Mousavi A, Gilani MM, Barooti E, Miratashi-Yazdi A, Dehghani A. Conservative treatment in early cervical cancer. International Journal of Biomedical Science. 2013;9(3):123-8.

15. Kim WY, Chang SJ, Chang KH, Yoo SC, Ryu HS. Conservative management of stage IA1 squamous cell carcinoma of the cervix with positive resection margins after conization. International Journal of Gynecology and Obstetrics. 2010;109(2):110-2.

16. Kokka F, Bryant A, Brockbank E, Jeyarajah A. Surgical treatment of stage IA2 cervical cancer. The Cochrane database of systematic reviews. 2014;5:CD010870.

17. Kolstad P. Follow-up study of 232 patients with Stage Ia1 and 411 patients with stage Ia2 squamous cell carcinoma of the cervix (microinvasive carcinoma). Gynecologic Oncology. 1989;33(3):265-72.

18. Kong T, Son J, Paek J, Chang S, Chang K, Ryu H. Outcomes of cold knife conization according to the margin involvement in high-grade lesions and microinvasive squamous cell carcinoma of the uterine cervix. Gynecologic Oncology. 2013;130(1):e53-e4.

19. Lee SJ, Kim WY, Lee JW, Kim HS, Choi YL, Ahn GH, et al. Conization using electrosurgical conization and cold coagulation for International Federation of Gynecology and Obstetrics stage IA1 squamous cell carcinomas of the uterine cervix. International Journal of Gynecological Cancer. 2009;19(3):407-11.

20. McHale MT, Le TD, Burger RA, Gu M, Rutgers JL, Monk BJ. Fertility sparing treatment for in situ and early invasive adenocarcinoma of the cervix. Obstetrics and Gynecology. 2001;98(5):726-31.

21. Monaghan JM. The management of microinvasive carcinoma of the cervix. CME Journal of Gynecologic Oncology. 2000;5(1):81-4.

22. Morris M. Management of stage IA cervical carcinoma. Journal of the National Cancer Institute Monographs. 1996(21):47-52.

23. Morris M, Mitchell MF, Silva EG, Copeland LJ, Gershenson DM. Cervical conization as definitive therapy for early invasive squamous carcinoma of the cervix. Gynecologic Oncology. 1993;51(2):193-6.

24. Nam JH, Kim SH, Kim JH, Kim YM, Kim YT, Mok JE. Nonradical treatment is as effective as radical surgery in the management of cervical cancer stage IA1. International Journal of Gynecological Cancer. 2002;12(5):480-4.

25. Ostor AG, Rome RM. Micro-invasive squamous cell carcinoma of the cervix: A clinico- pathologic study of 200 cases with long-term follow-up. International Journal of Gynecological Cancer. 1994;4(4):257-64.

26. Papakonstantinou K, Kyrgiou M, Lyons D, Soutter WP, Ghaem-Maghami S. Management of stage Ia1 squamous cervical cancer and the importance of excision margins: a retrospective study of long-term outcome after 25 years of follow-up. Am J Obstet Gynecol. 2014;211(6):625 e1-6.

27. Paraskevaidis E, Kitchener HC, Kalantaridou SN, Agnanti N, Lolis DE. Large loop conization for early invasive cervical cancer. International Journal of Gynecological Cancer. 1997;7(2):95-9.

28. Ramirez PT, Pareja R, Rendon GJ, Millan C, Frumovitz M, Schmeler KM. Management of low-risk early-stage cervical cancer: Should conization, simple trachelectomy, or simple hysterectomy replace radical surgery as the new standard of care? Gynecologic Oncology. 2014;132(1):254-9.

29. Raspagliesi F, Ditto A, Solima E, Quattrone P, Fontanelli R, Zanaboni F, et al. Microinvasive squamous cell cervical carcinoma. Critical Reviews in Oncology/Hematology. 2003;48(3):251-61.

30. Reade CJ, Eiriksson LR, Covens A. Surgery for early stage cervical cancer: How radical should it be. Gynecologic Oncology. 2013;131(1):222-30.

31. Rodriguez Oliver A, Hernandez F, Rojas R, Gomez T, Menjon S, Herruzo A. Treatment and follow-up of women with microinvasive carcinoma of cervix. Oncologia. 1994;17(7):31-5.

32. Schorge JO, Lee KR, Sheets EE. Prospective management of stage IA1 cervical adenocarcinoma by conization alone to preserve fertility: A preliminary report. Gynecologic Oncology. 2000;78(2):217-20.

33. Smrkolj S, Erzen M, Rakar S. The conservative management of patients with microinvasive cervical cancer. International Journal of Gynecology and Obstetrics. 2009;107:S343-S4.

34. Smrkolj S, Meglic L, Kosir Pogacnik R. Clinical outcome of patients with microinvasive adenocarcinoma of the uterine cervix. International Journal of Gynecological Cancer. 2013;23(8):788.

35. Sopracordevole F, Canzonieri V, Giorda G, De Piero G, Lucia E, Campagnutta E. Conservative treatment of microinvasive adenocarcinoma of uterine cervix: Long-term follow-up. Journal of Lower Genital Tract Disease. 2012;16(4):381-6.

36. Sopracordevole F, Chiossi G, Barbero M, Cristoforoni P, Ghiringhello B, Frega A, et al. Surgical approach and long-term clinical outcome in women with microinvasive cervical cancer. Anticancer Research. 2014;34(8):4345-50.

37. Spoozak L, Lewin S, Burke W, Deutsch I, Sun X, Herzog T, et al. Is there a microinvasive cervical adenocarcinoma? Gynecologic Oncology. 2011;120:S108-S9.

38. Spoozak L, Lewin SN, Burke WM, Deutsch I, Sun X, Herzog TJ, et al. Microinvasive adenocarcinoma of the cervix. American Journal of Obstetrics and Gynecology. 2012;206(1):80.e1-.e6.

39. Vavra N, Nagele F, Rosen A, Klein M, Barrada M, Buxbaum P, et al. Conisation in cervical carcinoma stage Ia. Geburtshilfe und Frauenheilkunde. 1994;54(7):397-400.

40. Winter R. Conservative surgery for microinvasive carcinoma of the cervix. Journal of Obstetrics and Gynaecology Research. 1998;24(6):433-6.

41. Wright JD, NathavithArana R, Lewin SN, Sun X, Deutsch I, Burke WM, et al. Fertility-conserving surgery for young women with stage IA1 cervical cancer: safety and access. Obstet Gynecol. 2010;115(3):585-90.

42. Yaegashi N, Sato S, Inoue Y, Noda K, Yajima A. Conservative surgical treatment in cervical cancer with 3 to 5 mm stromal invasion in the absence of confluent invasion and lymph-vascular space involvement. Gynecologic Oncology. 1994;54(3):333-7.

43. Yamaguchi H, Ueda M, Kanemura M, Izuma S, Nishiyama K, Tanaka Y, et al. Clinical efficacy of conservative laser therapy for early-stage cervical cancer. International Journal of Gynecological Cancer. 2007;17(2):455-9.

44. Zarchi MK, Mousavi A, Malekzadeh M, Dehghani A. Conservative treatment in young patients with cervical cancer: A review. International Journal of Gynecological Cancer. 2011;21(11):49.

45. Baalbergen A, Smedts F, Helmerhorst TJ. Conservative therapy in microinvasive adenocarcinoma of the uterine cervix is justified: an analysis of 59 cases and a review of the literature. International journal of gynecological cancer : official journal of the International Gynecological Cancer Society. 2011;21(9):1640-5.

46. Yahata H, Sonoda K, Yasunaga M, Ohgami T, Kawano Y, Kaneki E, et al. Surgical treatment and outcome of early invasive adenocarcinoma of the uterine cervix (FIGO stage IA1). Asia-Pacific Journal of Clinical Oncology. 2017.

47. Tomao F, Maruccio M, Preti EP, Boveri S, Ricciardi E, Zanagnolo V, et al. Conization in Early Stage Cervical Cancer: Pattern of Recurrence in a 10-Year Single-Institution Experience. Cell death discovery. 2017;27(5):1001-8.

48. Hartman CA, Teixeira JC, Barbosa SB, Figueiredo SM, Andrade LALDA, Bastos JFB. Analysis of Conservative Surgical Treatment and Prognosis of Microinvasive Squamous Cell Carcinoma of the Cervix Stage IA1: Results of Follow-Up to 20 Years. International Journal of Gynecological Cancer. 2017;27(2):357-63.

49. Slama J, Cerny A, Dusek L, Fischerova D, Zikan M, Kocian R, et al. Results of less radical fertility-sparing procedures with omitted parametrectomy for cervical cancer: 5 years of experience. Gynecologic oncology. 2016;142(3):401-4.

50. Yim GW, Kim SW, Nam EJ, Kim S, Kim HJ, Kim YT. Surgical outcomes of robotic radical hysterectomy using three robotic arms versus conventional multiport laparoscopy in patients with cervical cancer. Yonsei Med J. 2014;55(5):1222-30.

51. Bratila E, Bratila CP, Coroleuca CB. Radical Vaginal Trachelectomy with Laparoscopic Pelvic Lymphadenectomy for Fertility Preservation in Young Women with Early-Stage Cervical Cancer. The Indian journal of surgery. 2016;78(4):265-70.

52. Smith AL, Frumovitz M, Schmeler KM, dos Reis R, Nick AM, Coleman RL, et al. Conservative surgery in early-stage cervical cancer: what percentage of patients may be eligible for conization and lymphadenectomy? Gynecol Oncol. 2010;119(2):183-6.

53. Nick AM, Frumovitz MM, Soliman PT, Schmeler KM, Ramirez PT. Fertility sparing surgery for treatment of early-stage cervical cancer: open vs. robotic radical trachelectomy. Gynecol Oncol. 2012;124(2):276-80.

54. Abu-Rustum NR, Sonoda Y. Fertility-sparing radical abdominal trachelectomy for cervical carcinoma. Gynecologic Oncology. 2007;104(2 SUPPL.):56-9.

55. Achimas-Cadariu P, Traila A, Lancrajan I, Vlad C, Puscas E, Irimie A. The assessment of a simpler cold knife conization technique for the diagnosis and treatment of cervical dysplasia or microinvasive carcinoma. International Journal of Gynecological Cancer. 2013;23(8):679.

56. Al-Niaimi AN, Kushner DM. Cervical cancer recurrence after minimally invasive radical trachelectomy (MIRT) surgery. A review of the world's experience. Journal of Minimally Invasive Gynecology. 2011;18(6):S56-S7.

57. Andersen ES, Husth M, Joergensen A, Nielsen K. Laser conization for microinvasive carcinoma of the cervix. Short-term results. International Journal of Gynecological Cancer. 1993;3(3):183-5.

58. Andersen ES, Nielsen K, Pedersen B. Combination laser conization as treatment of microinvasive carcinoma of the uterine cervix. European Journal of Gynaecological Oncology. 1998;19(4):352-5.

59. Benedetti Panici P, Palaia I, Basile S, Perniola G, Sansone M, Gradinaru N, et al. Conservative approaches in early stages of cervical cancer. Gynecologic Oncology. 2007;107(1 SUPPL.):S13-S5.

60. Burghardt E, Girardi F, Lahousen M, Pickel H, Tamussino K. Microinvasive carcinoma of the uterine cervix (International Federation of Gynecology and Obstetrics Stage IA). Cancer. 1991;67(4):1037-45.

61. Chang MC, Liu HC. The value of conization for diagnosis and treatment of in situ and microinvasive carcinoma of uterine cervix. A pathological study of 569 conization cases. Chinese Medical Journal (Taipei). 1980;27(1):427-31.

62. Creasman WT, Fetter BF, Clarke-Pearson DL, Kaufmann L, Parker RT. Management of stage IA carcinoma of the cervix. Am J Obstet Gynecol. 1985;153(2):164-72.

63. Dhellemmes C. Microinvasive carcinomas of the cervix, evaluation of 9 years of conizations. Journal de gyn?cologie, obst?trique et biologie de la reproduction. 1986;15(8):1109-12.

64. Falconer AD. Conservative surgery for early stage cervical carcinoma. Reviews in Gynaecological and Perinatal Practice. 2006;6(1-2):1-11.

65. Ivanov S, Kornovski J, Kovachev E, Hinev A, Tzonev A, Ismail E, et al. [Our and foreign experience in modern surgical treatment of cervical adenocarcinoma stage T(1A1)]. Akusherstvo i ginekologii{combining double inverted breve}a. 2013;52 Suppl 1:23-5.

66. Kanayama S, Nakagawa E, Ueno S, Muraji M, Wakahashi S, Sudo T, et al. Outcomes of laser conization for cervical intraepithelial neoplasia 2-3 and microinvasive cervical cancer. World Journal of Oncology. 2014;5(2):62-7.

67. Koliopoulos G, Sotiriadis A, Kyrgiou M, Martin-Hirsch P, Makrydimas G, Paraskevaidis E. Conservative surgical methods for FIGO stage IA2 squamous cervical carcinoma and their role in preserving women's fertility. Gynecologic Oncology. 2004;93(2):469-73.

68. Lee JY, Kim HS, Kim K, Chung HH, Kim JW, Park NH, et al. Safety of less aggressive surgery for stage IA1 squamous cell carcinoma of the cervix. Journal of Obstetrics and Gynaecology Research. 2014;40(5):1382-8.

69. Lu Q, Liu C, Zhang Z. Total laparoscopic radical trachelectomy in the treatment of early-stage cervical cancer: Review of technique and outcomes. Current Opinion in Obstetrics and Gynecology. 2014;26(4):302-7.

70. Milliken DA, Shepherd JH. Fertility preserving surgery for carcinoma of the cervix. Current Opinion in Oncology. 2008;20(5):575-80.

71. Ottosen C. Trachelectomy for cancer of the cervix: Dargent's operation. Vaginal hysterectomy for early cancer of the cervix stage IA1 and CIN III. Best Practice and Research: Clinical Obstetrics and Gynaecology. 2011;25(2):217-25.

72. Pahisa J, Alonso I, Torne A. Vaginal approaches to fertility-sparing surgery in invasive cervical cancer. Gynecologic Oncology. 2008;110(3 SUPPL.2):S29-S32.

73. Qudah S, Azzam OA, Athamneh T, Baum S, Solomayer EF, Hammadeh M. Trachelectomy in early cervical cancer. International Journal of Women's Health and Reproduction Sciences. 2015;3(2):111-4.

74. Reynolds EA, Aletti GD, Cliby WA. Microinvasive adenocarcinoma of the cervix: Evaluation of the efficacy of conservative management. Gynecologic Oncology. 2009;112(2):S48-S9.

75. Reynolds EA, Tierney K, Keeney G, Felix J, Roman L, Cliby WA. Microinvasive adenocarcinoma of the uterine cervix is amenable to conservative management. Journal of Pelvic Medicine and Surgery. 2010;16(2):S9.

76. Shepherd JH, Milliken DA. Conservative Surgery for Carcinoma of the Cervix. Clinical Oncology. 2008;20(6):395-400.

77. Takeda N, Suzuki T, Suzuki M, Matsuura M, Tanaka R, Fujii M, et al. Evaluation of cervical conization as a definitive treatment for microinvasive cervical carcinoma and cervical intraepithelial neoplasia grade 3. Archives of Gynecology and Obstetrics. 2012;285(2):453-7.

78. Tseng CJ, Chang CC, Tseng CC, Hou HC, Wang CB, Chen CH, et al. Loop conization for the treatment of microinvasive carcinoma of the cervix. International Journal of Gynecological Cancer. 2006;16(4):1574-8.

79. Tseng CJ, Horng SG, Soong YK, Hsueh S, Hsieh CH, Lin HW. Conservative conization for microinvasive carcinoma of the cervix. Am J Obstet Gynecol. 1997;176(5):1009-10.

80. Ueda M, Ueki K, Kanemura M, Izuma S, Yamaguchi H, Terai Y, et al. Conservative excisional laser conization for early invasive cervical cancer. Gynecologic Oncology. 2004;95(1):231-4.

81. Ueki M. Conservative therapy for microinvasive carcinoma of the uterine cervix. Gynecologic Oncology. 1994;53(1):109-13.

82. Yahata T, Nishino K, Kashima K, Sekine M, Fujita K, Sasagawa M, et al. Conservative treatment of stage IA1 adenocarcinoma of the uterine cervix with a long-term follow-up. International Journal of Gynecological Cancer. 2010;20(6):1063-6.

83. Yoshinaga M, Hamada T, Orita Y, Yoshitomi Shintomo N, Matsuo T, Tsuji T, et al. Clinical outcome of stage Ia1 squamous cell carcinoma of the uterine cervix and pathological findings of initial conization. The journal of obstetrics and gynaecology research. 2011;37(11):1645-9.

84. Yousefi Z, Kazemianfar Z, Kadghodayan S, Hasanzade M, Kalantari M, Mottaghi M. Less radical surgery for patient with early-stage cervical cancer. Iranian Red Crescent Medical Journal. 2013;15(7):617-9.

85. Zeng SY, Liang MR, Li LY, Li L, Jiang W, Zhong ML. [Application of transvaginal external fascia trachelectomy in the treatment of CIN and micro-invasive cervical cancer]. Zhonghua zhong liu za zhi [Chinese journal of oncology]. 2013;35(7):543-6.

86. Reynolds EA, Tierney K, Keeney GL, Felix JC, Weaver AL, Roman LD, et al. Analysis of outcomes of microinvasive adenocarcinoma of the uterine cervix by treatment type. Obstetrics and gynecology. 2010;116(5):1150-7.

87. Chakalova G, Karag'ozov A. Organ-preserving operations on patients with microinvasive carcinoma of the cervix uteri. Akusherstvo i ginekologiia. 1991;30(3):51-3.

88. Andikyan V, Khoury-Collado F, Denesopolis J, Sandadi S, Park K, Brown C, et al. Cervical conization and sentinel lymph node mapping in the treatment of stage I cervical cancer: Is less enough? Gynecologic Oncology. 2013;130(1):e50.

89. Plante M, Gregoire J, Renaud MC, Roy M. Simple vaginal trachelectomy and laparoscopic lymph node evaluation in patients with low risk early-stage cervical cancer. International Journal of Gynecological Cancer. 2012;22:E200.

90. Wright J, Nathavithrana R, Lewin S, Sun X, Deutsch I, Burke W, et al. Safety and access to fertility-conserving surgery for young women with stage IA1 cervical cancer. Gynecologic Oncology. 2010;116(3):S146.

91. Rob L, Charvat M, Robova H, Pluta M, Strnad P, Hrehorcak M, et al. Less radical fertility-sparing surgery than radical trachelectomy in early cervical cancer. International Journal of Gynecological Cancer. 2007;17(1):304-10.

92. Rob L, Robova H, Pluta M, Halaska MJ, Matecha J, Skapa P. Less radical surgery than radical trachelectomy or radical hysterectomy in patients with stage i cervical cancer. Gynecologic Oncology. 2014;133:66-7.

93. Plante M, Gregoire J, Renaud MC, Sebastianelli A, Grondin K, Noel P, et al. Simple vaginal trachelectomy in early-stage low-risk cervical cancer a pilot study of 16 cases and review of the literature. International Journal of Gynecological Cancer. 2013;23(5):916-22.

94. Willows K, Lennox G, Covens A. Fertility-sparing management in cervical cancer: balancing oncologic outcomes with reproductive success. Gynecologic oncology research and practice. 2016;3:9.

95. Kardakis S. Fertility-preserving surgery in patients with early stage cervical carcinoma. ISRN oncology. 2012;2012:817065.

96. Ramirez PT, Pareja R, Rendon GJ, Millan C, Frumovitz M, Schmeler KM. Management of low-risk early-stage cervical cancer: should conization, simple trachelectomy, or simple hysterectomy replace radical surgery as the new standard of care? Gynecol Oncol. 2014;132(1):254-9.
