# Supplementary material for: Can simple trachelectomy or conization show comparable survival rate compared with radical trachelectomy in IA1 cervical cancer patients with lymphovascular space invasion who wish to save fertility? A systematic review and guideline recommendation
Source: PLoS One. 2018 Jan 31;13(1):e0189847. doi: 10.1371/journal.pone.0189847 (PMC5791938; doi:10.1371/journal.pone.0189847)
Supplement: S2 Table — (DOC) [file pone.0189847.s004.doc]

**S2 Table. Newcastle–Ottawa Scale for the risk of bias and the quality assessment of included studies**

| Author | Year | Selection | | | | Comparability | Exposure | | | Total score |
| --- | --- | --- | --- | --- | --- | --- | --- | --- | --- | --- |
|  |  | Adequate definition of patient cases | Representativeness of patient cases | Selection of controls | Definition of controls | Control for important or additional factors | Ascertainment of exposure | Same method of ascertainment for participants | Nonresponse rate^*^ |  |
| Bekkers et al. | 2002 | ⋆ | ⋆ |  | ⋆ | ⋆ | ⋆ | ⋆ | ⋆ | 7 |
| Lee et al. | 2009 | ⋆ | ⋆ |  | ⋆ | ⋆ | ⋆ | ⋆ | ⋆ | 7 |

* One point was awarded when no significant difference was noted in the response rate between the two groups based on the Chi-square test (*P*<0.05).
