# Supplementary material for: Can simple trachelectomy or conization show comparable survival rate compared with radical trachelectomy in IA1 cervical cancer patients with lymphovascular space invasion who wish to save fertility? A systematic review and guideline recommendation
Source: PLoS One. 2018 Jan 31;13(1):e0189847. doi: 10.1371/journal.pone.0189847 (PMC5791938; doi:10.1371/journal.pone.0189847)
Supplement: S3 Table — (DOC) [file pone.0189847.s005.doc]

**S3 Table.** Seven standard criteria suggested by the Cochrane Effective Practice and Organisation of Care (EPOC) to evaluate the study quality for single-arm cohort studies

| Author | Year | Was the intervention independent of other changes? | Was the shape of the intervention effect pre-specified? | Was the intervention unlikely to affect data collection? | Was knowledge of the allocated interventions adequately prevented during the study? | Were incomplete outcome data adequately addressed? | Was the study free from selective outcome reporting? | Was the study free from other risks of bias? |
| --- | --- | --- | --- | --- | --- | --- | --- | --- |
| Rob et al. | 2008 | Low | Low | Low | Low | Low | Low | Low |
| Plante et al. | 2017 | Low | Low | Low | Low | Low | Low | Low |
| Andikyan et al. | 2014 | Low | Low | Low | Low | Low | Low | Low |
