# Supplementary material for: Can simple trachelectomy or conization show comparable survival rate compared with radical trachelectomy in IA1 cervical cancer patients with lymphovascular space invasion who wish to save fertility? A systematic review and guideline recommendation
Source: PLoS One. 2018 Jan 31;13(1):e0189847. doi: 10.1371/journal.pone.0189847 (PMC5791938; doi:10.1371/journal.pone.0189847)
Supplement: S4 Table — (DOC) [file pone.0189847.s006.doc]

**S4 Table.** An “evidence profile” by grading the quality of evidence according to the Grading of Recommendation, Assessment, Development and Evaluation (GRADE) system

| Outcomes | Illustrative comparative risks (95% CI) | Relative effect  (95% CI) | No of Participants  (studies) | Quality of the evidence  (GRADE) |
| --- | --- | --- | --- | --- |
| Follow-up: 5-6years | Not estimable | Not estimable | 64  (4 studies) | ⊕⊝⊝⊝  very low |
